# Low‐cost, handheld, multi‐pulse electroporators for simplified nucleic acid delivery in skin

**DOI:** 10.1002/btm2.70070

**Published:** 2025-09-08

**Authors:** Pankaj Rohilla, Erkan Azizoglu, Sion Park, Atharva Lele, Mark R. Prausnitz, Saad Bhamla

**Affiliations:** ^1^ School of Chemical & Biomolecular Engineering Georgia Institute of Technology Atlanta Georgia USA

**Keywords:** electroporation, in vivo transfection, microneedle electrodes, multi‐pulse piezoelectricity, naked mRNA/DNA delivery

## Abstract

Electroporation‐mediated delivery offers a promising alternative to carrier‐based nucleic acid delivery methods for vaccination and therapeutic applications. Carrier‐based systems like lipid nanoparticles and viral vectors often suffer from poor in vivo stability, immunogenicity, toxicity, and off‐target effects. To overcome the high cost, bulkiness, lack of portability, and painful administration of traditional electroporators, we developed the RotoPatch family of small, low‐cost, hand‐held piezoelectric electroporators that use microneedle electrodes for intradermal delivery of nucleic acids. Notably, these RotoPatch devices use a single rotary motion to administer multiple electroporation pulses through microneedle electrodes, that localize the electric field to the upper layers of the skin. In animals, RotoPatch facilitated greater intracellular uptake of firefly luciferase‐encoded mRNA in mice and green fluorescent protein‐encoded plasmid DNA in rats, as confirmed by bioluminescence and fluorescence imaging, respectively. RotoPatch produced similar in vivo expression as electroporation using a manually actuated, multi‐pulse piezoelectric electroporator (ePatch) and a battery‐powered, multi‐pulse electroporator (eIgniter). These findings highlight the potential of multi‐pulse piezoelectric microneedle electroporation for intradermal nucleic acid delivery as a platform for gene therapy and vaccination.

**Translational Impact Statement**

We developed low‐cost, portable, multi‐pulse piezoelectric electroporators for carrier‐free intradermal delivery of naked mRNA and plasmid DNA. By localizing the electric field to the upper skin using microneedle electrode arrays, these devices enhanced in vivo expression of reporter proteins. Motorized rotary actuation enabled rapid pulse delivery with controlled timing. These scalable, operator‐friendly electroporators offer an accessible platform for gene therapy and vaccination.

## 1 INTRODUCTION

Since the first demonstration of in vivo protein expression from mRNA and DNA delivered into the skin in 1990, both nucleic acids have steadily gained recognition as powerful therapeutic platforms.^1^ The recent success of mRNA‐based SARS‐CoV‐2 vaccines has further accelerated interest in nucleic acid–based therapies, expanding their scope to treat other infectious diseases, cancer, muscular and retinal dystrophies, and various genetic disorders.^2^, ^3^, ^4^ Approved DNA‐based vaccines, such as Zydus's ZyCov‐D for human use^5^ and multiple vaccines for use in animals,^6^ along with mRNA‐based human vaccines like Moderna's mRNA‐1273 (Spikevax),^7^ and Pfizer‐BioNTech's BNT162b2 (Comirnaty)^8^ have established a precedent for the safe and effective use of nucleic acid delivery at large scale. More recently, Moderna's mRNA‐1345, an mRNA vaccine against respiratory syncytial virus (RSV),^9^ was approved in the United States and Europe in 2024. Similarly, DNA‐based gene therapies such as Novartis' Zolgensma for spinal muscular atrophy^10^ and BioMarin's AAV5‐hFVIII‐SQ gene therapy for hemophilia A,^11^ highlight the ongoing clinical success of gene therapy.

Despite these advances, the intracellular delivery of nucleic acids has remained largely confined to viral^12^, ^13^, ^14^ vectors, including adenovirus (Ad), adeno‐associated virus (AAV), and lentivirus, and non‐viral vectors employing different mechanisms, such as lipid nanoparticles (LNPs), polymeric nanoparticles, and extracellular vesicles.^12^, ^13^, ^15^, ^16^, ^17^, ^18^, ^19^ However, both of these types of carriers have limitations, including immunogenicity, limited and expensive scalability, poor endosomal escape, toxicity, off‐target effects, and carrier degradation.^20^, ^21^, ^22^ These limitations have prompted increasing demand for carrier‐free methods for cytosolic delivery of naked nucleic acids.^23^ Another challenge for mRNA‐based delivery remains the requirement of ultra‐cold storage.^24^, ^25^

In contrast to carrier‐based nucleic acid delivery methods, electroporation was first introduced in the 1980s^26^ and shown to be highly effective for intracellular nucleic acid delivery. Short, high‐voltage pulses transiently permeabilize the cell membrane, enabling enhanced uptake. The efficiency and safety of electroporation are highly dependent on pulse parameters, including waveform type, duration, amplitude, and frequency.^27^

Early systems using exponential decay pulses quickly fall below the critical voltage threshold for permeabilization, with excess energy driving electrophoresis, Joule heating, and electrolysis.^27^ This limitation can be overcome with bipolar square‐wave pulses, which sustain the electric field above the voltage threshold throughout the pulse.^27^ More recently, microsecond‐scale oscillatory bipolar waveforms have further improved membrane permeabilization while preserving cell viability, likely due to transient sonication‐like effects and minimized membrane polarization.^28^, ^29^, ^30^ Additionally, bipolar pulses reduce electrolysis and muscle contractions, improving tolerability and supporting broader clinical use.

To maintain membrane integrity and ensure pore resealing, electric fields of 1–3 kV/cm have been used traditionally for microsecond‐duration pulses,^30^, ^31^, ^32^ while weaker fields (<1 kV/cm) are used for longer, millisecond‐duration pulses.^33^ Once delivered, mRNA is translated into protein in the cytoplasm, while plasmid DNA must enter the nucleus for transcription before protein expression. The resulting proteins can then elicit an immune response against infectious agents or serve therapeutic functions in treating diseases. For example, electroporation‐mediated delivery of nucleic acids has been shown to enhance the immune response in both infectious‐disease vaccination and cancer immunotherapy.^30^, ^34^

Several commercial electroporation platforms have demonstrated enhanced nucleic‐acid delivery in both preclinical and clinical settings. Devices such as IGEA's Cliniporator,^35^, ^36^ BTX's AgilePulse,^37^, ^38^ Ichor Medical Systems' TriGrid,^39^ and Inovio's CELLECTRA^40^ have been employed for DNA vaccine delivery and cancer immunotherapy, achieving enhanced transfection efficiency and robust antigen‐specific immune responses. However, high cost, bulky size, scalability, requirement of electricity, and painful administration have limited their clinical use.^41^, ^42^, ^43^

Recently, we developed an ultra‐low‐cost, piezoelectric‐based electroporation device (ePatch) for intradermal delivery of both mRNA and plasmid DNA, which achieved robust protein and gene expression, eliciting strong immune responses against SARS‐CoV‐2 with both mRNA‐ and plasmid‐based COVID‐19 vaccines.^30^, ^44^ By using a microneedle electrode array (MEA) that penetrates only into the upper layers of skin, ePatch was shown in human subjects to be virtually painless and avoided muscle twitching observed with conventional electroporation systems.^45^

Effective gene transfection typically requires 3–10 electric pulses to the skin to significantly enhance intracellular nucleic acid uptake.^30^, ^35^, ^37^, ^38^ However, this need for multiple pulses introduces limitations with the manually triggered ePatch, including user fatigue from repeated clicks, inconsistent inter‐pulse intervals, and variability in applied force and microneedle‐skin contact all of which can contribute to inconsistent delivery performance by a user‐dependent device.

To address these limitations and improve clinical usability, we developed RotoPatch, a novel motorized piezoelectric electroporation platform capable of delivering up to nine electric pulses with a single cap rotation. The family of RotoPatch pulsers builds upon the low‐cost piezoelectric pulse generation principle previously demonstrated with the manually triggered ePatch,^30^, ^46^ but replaces repeated clicking with a rotary‐driven mechanism to enable rapid, multi‐pulse delivery in a single actuation, improving ease of use and consistency.

In this study, we evaluated the feasibility of RotoPatch for intradermal delivery of nucleic acids in animal models, comparing its performance to both the ePatch and a battery‐powered, electrical pulser (eIgniter) that contains no moving parts. These electroporation platforms were evaluated based on their efficiency in delivering mRNA and plasmid DNA, as measured by in vivo protein and gene expression in rodents. We propose that the enhanced and sustained expression achieved with these frugal electroporation systems may support broader adoption of electroporation‐based delivery for vaccines and gene therapies as an alternative to carrier‐based systems.

## 2 MATERIALS AND METHODS

### 2.1 Device fabrication

#### 2.1.1 3D printing

The structural components and housing for both the manual and motorized RotoPatch versions were designed using computer‐aided design (CAD) software (Fusion 360, Autodesk, San Francisco, CA). Fabrication of these components was performed using fused deposition modeling. All printing tasks were performed using a custom‐built 3D printer (LDO Motors, Shenzhen, China) based on the Voron Trident (Voron Design, https://vorondesign.com). A high‐strength polylactic acid (PLA) filament (PolySonic PLA Pro, Polymaker, Missouri City, TX) was used as the printing material, selected for its ease of printing, enhanced toughness, and increased rigidity. Print files were prepared and sliced using OrcaSlicer software. A list of components of the devices used in this study is tabulated in Supplementary Table S1.

#### 2.1.2 Pulsers

The RotoPatch pulser was made using a piezoelectric crystal, stainless steel hammer, spring (spring constant ~230 N/m) harvested from a barbeque lighter, and 3D printed components. For the motorized RotoPatch, a NEMA 17 stepper motor (LDO Motors, Shenzen, China) controlled by an Arduino Uno microcontroller board (Arduino AG, Chiasso, Switzerland) was used to set an inter‐pulse interval of 300 ms. The pulsers were based on barbeque‐lighter igniters and were used as received for the ePatch, as described previously,^46^ and the eIgniter (Grisun, China).

#### 2.1.3 Microneedle electrode array

All electroporators were connected to the same type of MEA housed in a 3D‐printed holder containing six rows of stainless‐steel microneedle electrodes fabricated by chemical etching (Tech Etch, Plymouth, MA). Each microneedle row was 50 μm thick and consisted of nine microneedles, each measuring 650 μm in length with a tip‐to‐tip spacing of 790 μm. The inter‐array spacing between adjacent electrode arrays with opposite polarities was maintained at 650 μm. In this way, the complete MEA contained 54 microneedles.

### 2.2 Characterization

Voltage and current profiles from different pulsers were recorded using a digital oscilloscope (SDS 1202X‐E, Siglent, Shenzhen, China) at a sampling rate of 25 MHz. Voltage measurements made across an open circuit were obtained using a 1000X high‐voltage probe connected between the oscilloscope and the pulser (Supplementary Figure S1). Voltage and current measurements were made across skin by pressing a MEA into porcine skin ex vivo (Pel‐Freeze, Rogers, AR), measuring voltage across the skin, and determining current by measuring the voltage across a 100‐Ω resistor placed in series with the skin; the current was calculated as the voltage drop across the resistor divided by resistance.

Transient force profiles during pulser actuations were measured using a single‐point load cell (20 kg, RSP1‐020M‐A CO1, Loadstar Sensors, Fremont, CA), with data collected at a 10 kHz sampling rate using the Loadstar software.

### 2.3 Animals

All animal experiments were conducted in accordance with ethical approval from the Institutional Animal Care and Use Committee (IACUC, protocol # A100427) of the Georgia Institute of Technology. BALB/c mice (female, 7–18 weeks old) and adult Wistar rats (female, 6–8 weeks old) were obtained from Charles River Laboratories (Charles River, Wilmington, MA) and housed in a pathogen‐free facility at the Georgia Institute of Technology under a 12 h light/12 h dark schedule (lights on at 07:00, off at 19:00), with water and chow available ad libitum. For this feasibility study, we did not perform power calculations to determine sample size or use an animal randomization schedule. Investigators were not blind to the experimental groups. The ARRIVE guideline summary for the animal study is provided in the supplementary information.

### 2.4 Intradermal injections and electroporation

Mice and rats were prepared under anesthesia 1 day prior to ID injections by removing hair on their dorsal skin with clippers, followed by a 1–2‐min application of depilatory cream (Nair, Church & Dwight, Ewing, NJ), which was then cleaned off with lukewarm water. Animals were anesthetized in an induction charged with 5% isoflurane in O_2_ by isoflurane vaporizer (SurgiVet Model 100, Smiths Medical, Dublin, OH), then fitted with a standard rodent mask and maintained under general anesthesia with 1%–2% isoflurane during the procedures.

Twenty microliters of phosphate‐buffered saline (PBS) free of Ca^2+^ and Mg^2+^ and containing firefly‐luciferase encoding N1‐methylpseudouridine (m^1^Ψ)‐modified naked mRNA (0.25 μg/μL, Genscript, Piscataway, NJ) with RNase inhibitor (1 U/μL, RNasin Plus; Promega, WI), or gWiz‐GFP plasmid (2.5 μg/μL, Aldevron, Freiburg, Germany) was intradermally injected using a 31G insulin syringe (Easytouch U‐100, MHC medical products, Fairfield, OH), forming a visible bleb at the skin surface of mice and rats, respectively (Supplementary Figure S2). Immediately after the injection, 10 electric pulses were administered through MEA insertion at the bleb site. For ePatch and the eIgniter, pulses were triggered manually by clicking the buttons on the pulsers, with ~1 s between pulses, respectively. For RotoPatch, an Arduino‐controlled script automated pulse delivery with a 300 ms interval between successive pulses (Supplementary Figure S3).

### 2.5 IVIS imaging

Bioluminescence and fluorescence imaging were used, respectively, to quantify the expression kinetics of firefly luciferase mRNA and green fluorescent protein (GFP) plasmid in mice and rats. Prior to bioluminescence imaging, mice were injected intraperitoneally with D‐luciferin (200 μL, 15 mg/mL; Gold Biotechnology, Olivette, MO) and were imaged within 15–25 min using the IVIS Lumina II system (PerkinElmer, Waltham, MA). Total photon flux (bioluminescence, in mice) and radiant efficiency (fluorescence, in rats) were quantified using Living Image Software (v4.3.1, Caliper Life Sciences, Hopkinton, MA) within a fixed circular region of interest at each ID injection site. Imaging was conducted on multiple days post‐injection to measure the expression kinetics of naked mRNA and GFP‐plasmid with and without electroporation, with ID injected PBS as a negative control. To quantify protein and gene expression from mRNA and plasmid DNA, we calculated the area under the expression curve using the trapezoidal rule in MATLAB (Supplementary Figure S4).

### 2.6 Statistical methods

Statistical analysis was performed in Prism 5.3 software (GraphPad, San Diego, CA). The Shapiro–Wilk test was performed to check the normality of the in vivo protein expression data for different study groups. For mRNA data, one group did not meet the assumption of normality; therefore, the Friedman test followed by Dunn's post‐hoc multiple comparisons was used for statistical comparison. In contrast, for normally distributed plasmid DNA data, repeated measures analysis with Dunnett's multiple comparisons test was used. Statistical significance was established with *p*‐values less than 0.05.

## 3 RESULTS

### 3.1 Design, fabrication and operation of RotoPatch

**FIGURE 1:**
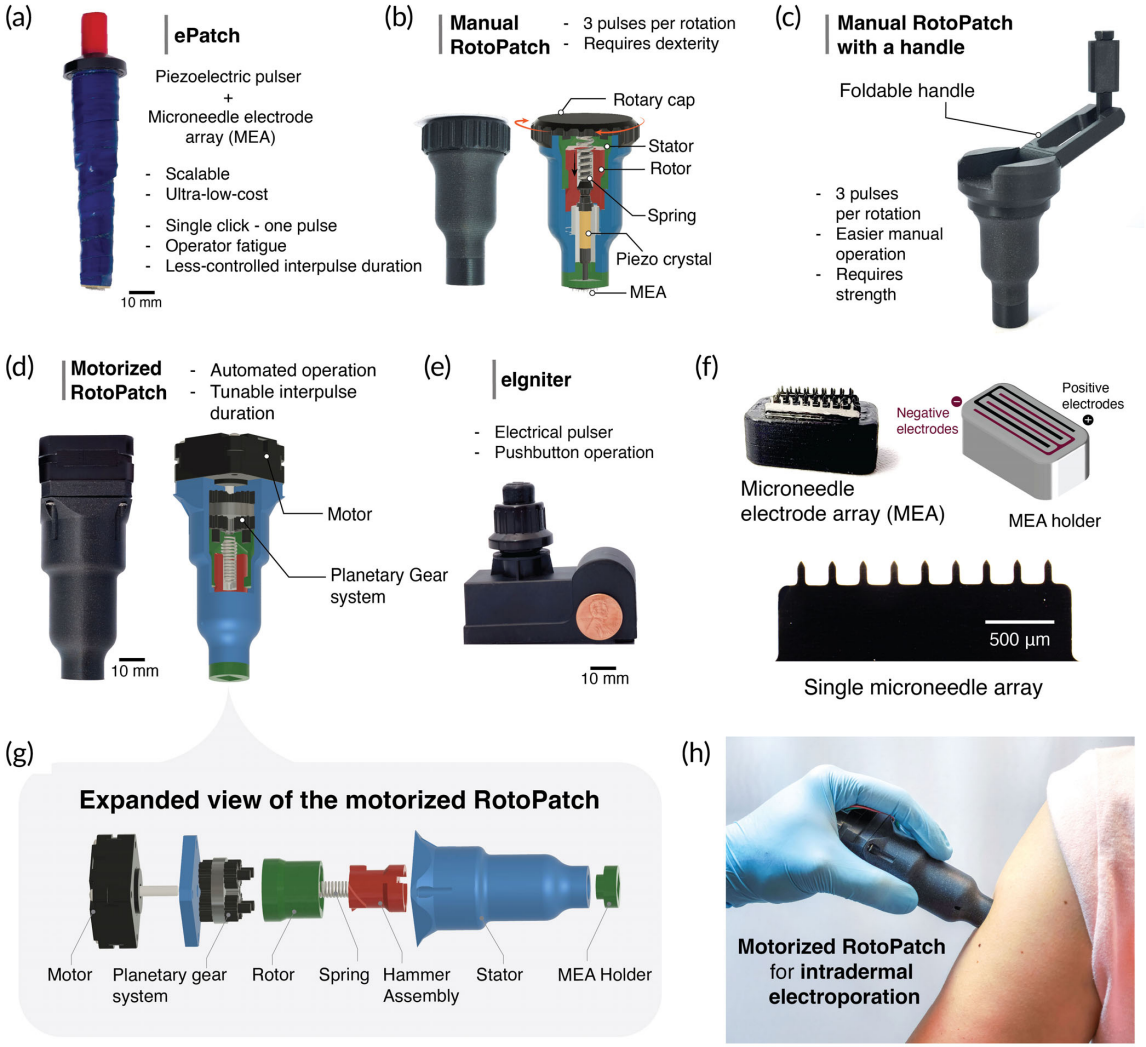
Design evolution of RotoPatch pulsers. (a) Original ePatch with single‐pulse actuation of piezoelectric pulses. (b) Manual RotoPatch with manual rotary cap activation of multiple piezoelectric pulses. (c) Manual RotoPatch with a handle with rotational activation of multiple piezoelectric pulses using a foldable handle. (d) Motorized RotoPatch with automated rotational activation of multiple piezoelectric pulses using a small electric motor. (e) eIgniter with electronically controlled activation of battery‐powered pulses. (f) A microneedle electrode array (MEA), a CAD rendering of the MEA holder showing slots for both positive and negative electrodes, and a single row of microneedle electrodes. (g) CAD renderings of key components of the motorized RotoPatch, including the motor, planetary gear system, rotor, stator, spring, hammer assembly, and MEA holder. (h) Demonstration of the motorized RotoPatch being applied to a patient's upper arm.

We previously developed ePatch as a means to generate electroporation pulses using a small, low‐cost, hand‐held device that leverages the piezoelectric element of a barbeque lighter to produce electric pulses^30^ (Figure 1a). While effective, the ePatch is only able to administer one electric pulse for each manual actuation of the device. Because electroporation often benefits from giving multiple pulses, a user needs to repeatedly actuate ePatch each time a pulse is given. The force of actuation to energize the piezoelectric element to produce an electrical discharge when using ePatch is also relatively high, leading to possible user fatigue.

To address these limitations, we developed RotoPatch (Figure 1b–d), which consists of 3D‐printed components and a casing that houses a spring, a piezoelectric crystal, and a MEA (Figure 1f). Its rotational actuation mechanism enables pulse generation with a low tangential force, nearly 10 times lower than the normal force required to actuate a single click of the ePatch to generate a single pulse (Supplementary Figure S5), rendering the RotoPatch more user‐friendly for clinical operators.

The pulse generation of the RotoPatch involves conversion of rotational energy into repeated translational strikes through a simple mechanical sequence (Supplementary Figure S6). As the cap and rotor rotate, interfacing angled ridges on both the rotor and the hammer assembly cause the hammer assembly to lift vertically, compressing the spring until it reaches the ridge peak. Immediately after passing the peak, the hammer is suddenly released and strikes a metal pin positioned above the piezoelectric crystal, generating an electric pulse. This sequence repeats multiple times per rotation, producing a consistent train of pulses.

We developed a family of three distinct versions of the RotoPatch: (i) a manual RotoPatch, operated by hand rotation similar to loosening the lid on a jar (Figure 1b); (ii) a manual RotoPatch with a handle, operated by rotation of a foldable handle similar to a manual coffee grinder handle (Figure 1c); and (iii) a motorized RotoPatch, powered by a stepper motor controlled by a microcontroller that is activated with a simple button push (Figure 1d, g, h). The manual RotoPatch devices enable electricity‐free use with a simple, low‐cost design, while the motorized RotoPatch offers precise rate control and no need for user strength or dexterity. As an alternative design, we also included the eIgniter, which has fully electronic control and production of electric pulses actuated by pushbutton (Figure 1e).

In all RotoPatch designs, rotational input is converted into sequential hammer strikes via a spring‐latch mechanism, enabling consistent high‐voltage pulse generation. The manual versions use a rotary cap to facilitate repeated impacts, while the motorized version achieves programmable, repeatable actuation. The number of pulses delivered per rotation is configurable by design; prototypes featured 3 pulses/rotation (optimized for low rotational torque, suitable for motorized operation or handle‐free manual use) and 9 pulses/rotation (maximizing the pulse count per rotation for handled manual use, thereby reducing total required rotation). Increasing the pulse count per rotation necessitated steeper internal ridge geometry and consequently required higher input torque. This design flexibility highlights RotoPatch as a tunable, low‐cost, and scalable alternative to conventional electroporation systems.

For this study, electroporation systems consisted of a pulse generator (Figure 1a–e) and a MEA (Figure 1f), which comprised a 3D‐printed holder containing six rows of stainless‐steel microneedle electrodes, fabricated using a chemical etching technique. Each microneedle electrode was 50 μm thick and was arranged in a row of nine microneedles, each measuring 650 μm in length with a tip‐to‐tip spacing of 790 μm. The inter‐array spacing between adjacent electrode arrays was maintained at 650 μm. This MEA configuration enabled the electric field generated by the pulser devices to be localized within the epidermis and upper dermis, targeting skin‐resident immune cells such as Langerhans and dermal dendritic cells for enhanced vaccination.

Both piezoelectric pulsers (ePatch and RotoPatch) involve moving parts leading to noisy operation and wear and tear of mechanical parts over time. Therefore, we developed another barbeque lighter‐inspired electrical pulser, eIgniter (Figure 1e), as a fully automatic electronic pulse generator. The eIgniter consists of an ignition module (transformer and two electrodes), a 9 V battery, and a pushbutton.

### 3.2 Electrical characterization of RotoPatch, ePatch, and eIgniter

**FIGURE 2:**
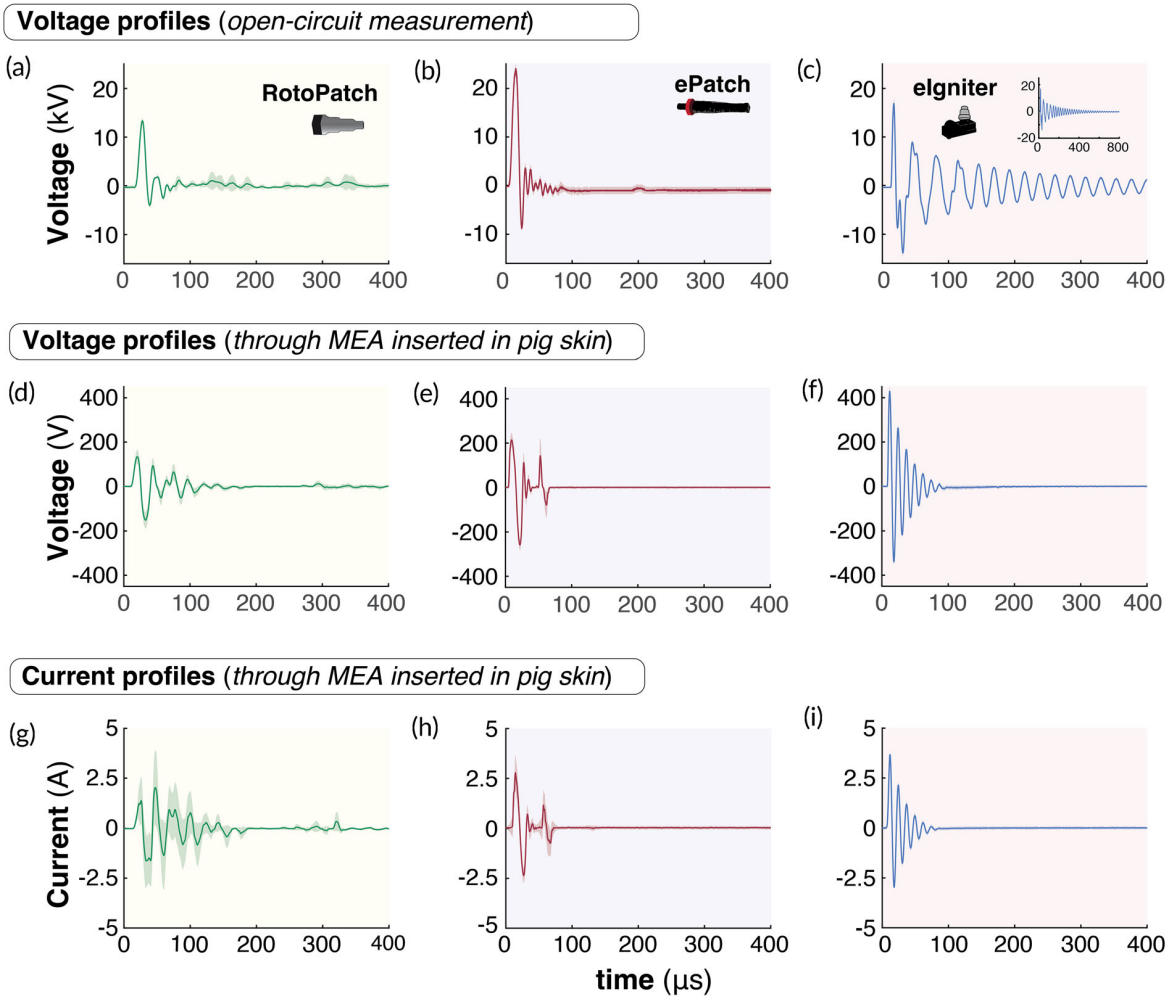
Electrical characterization of RotoPatch, ePatch, and eIgniter. Open‐circuit voltage profiles generated by (a) RotoPatch, (b) ePatch, and (c) eIgniter during a single pulse discharge. Voltage profiles were recorded across microneedle electrode arrays (MEAs) inserted into pig skin ex vivo for (d) RotoPatch, (e) ePatch, and (f) eIgniter during a single pulse discharge. Corresponding current profiles through MEAs inserted into pig skin for (g) RotoPatch, (h) ePatch, and (i) eIgniter during a single pulse discharge. We used motorized RotoPatch for electrical characterization. Shaded regions represent standard deviations from the mean for *n* = 4.

We characterized the voltage profiles generated by the RotoPatch, ePatch, and eIgniter to compare their performance. In open‐circuit measurements, RotoPatch and ePatch pulsers generated bipolar oscillatory voltage waveforms with post‐pulse ringing, while the eIgniter pulser generated underdamped oscillatory waveforms (Figure 2a–c). RotoPatch generated positive and negative voltage peaks of 13.3 ± 0.3 kV (~11 μs) and −3.7 ± 0.2 kV (~22 μs), respectively (Figure 2a), which were significantly lower than those of the ePatch (24.1 ± 7.1 kV at ~11 μs and −9.0 ± 0.4 kV at ~20 μs, *p* <0.05, Figure 2b), and significantly higher than those of the eIgniter (16.6 ± 0.7 kV at ~4 μs and −13.6 ± 0.4 kV at ~19.3 μs, *p* <0.05, Figure 2c). The pulse decay times for the RotoPatch, ePatch, and eIgniter pulsers were 53.68 ± 0.47 μs, 73.93 ± 0.23 μs, and 668.03 ± 3.70 μs, respectively. The open‐circuit voltage outputs from the motorized and manual RotoPatch devices were similar (Supplementary Figure S7), indicating that the mechanism of rotational actuation did not affect the electrical performance of the motorized versus manual RotoPatch.

We also performed voltage measurements of RotoPatch, ePatch, and eIgniter administered to ex vivo porcine skin via the MEA. Compared to open‐circuit measurements, all devices showed reduced voltage amplitudes due to discharge across a closed circuit through skin (Figure 2d–f). The eIgniter produced the highest mean voltage peaks (438.8 ± 25.84 V and−335.33 ± 19.56 V, *p* <0.05, Figure 2f), followed by ePatch (228.20 ± 33.62 V and −262.98 ± 49.32 V, *p* <0.05, Figure 2e) and RotoPatch (141.98 ± 46.28 V and −160.07 ± 50.92 V, *p* <0.05, Figure 2d), with decay times spanning 78–132 μs. The voltage peaks observed for ePatch were consistent with previously reported measurements.^45^

Companion current measurements through porcine skin showed similar waveforms to the voltage outputs (Figure 2g–i). The eIgniter produced the highest peak currents (3.67 ± 0.07 A, and −2.97 ± 0.09 A, *p* <0.05, Figure 2i), followed by ePatch (2.78 ± 0.86 A and −2.36 ± 0.36 A, *p* <0.05, Figure 2h), and RotoPatch (2.02 ± 1.87 A and −2.11 ± 0.29 A, *p* <0.05, Figure 2g). The corresponding apparent skin impedances (i.e., the voltage divided by the current during the pulses) were 123.1 ± 7.7 Ω for eIgniter, 84.0 ± 14.5 Ω for ePatch, and 52.5 ± 18.9 Ω for RotoPatch.

Weak voltage and current signals were seen at ~300 μs when using RotoPatch (Figure 2a, d, g). We hypothesized that this was due to the hammer rebounding off the piezoelectric crystal and then striking the crystal again with lesser force before retraction. High‐speed video imaging of RotoPatch operation confirmed this hypothesis, showing a second hammer strike ~300 μs after the first one (Supplementary Video S1). While a similar second strike of the hammer was seen in video of the ePatch, we did not see evidence of it in the voltage and current profiles for ePatch (Figure 2b, e, h), perhaps because the second strike was too weak. Because there is no hammer strike involved in eIgniter operation, there would be no mechanism for this effect.

Electroporation for delivering nucleic acids such as mRNA and DNA in vaccination and therapeutic applications typically requires electric fields in the range of ~1–3 kV/cm when employing microsecond‐long pulses.^30^, ^45^ We calculated the nominal electric field strength generated by RotoPatch, ePatch, and eIgniter when pulsing pig skin using MEAs by dividing the peak voltage by the spacing between microneedle anode and cathode electrodes, which were approximately 2.8, 3.6, and 7.15 kV/cm, respectively. These values are roughly in the target range, although the eIgniter voltage was higher.

It is worth noting that the electric field strength was probably not constant throughout the treated region of skin, with increased field strength immediately next to microneedle electrode tips and edges due to their curvature and decreased field strength between microneedle electrodes of the same polarity (i.e., between microneedles in the same row).

### 3.3 RotoPatch enhancement of intradermal mRNA expression

**FIGURE 3:**
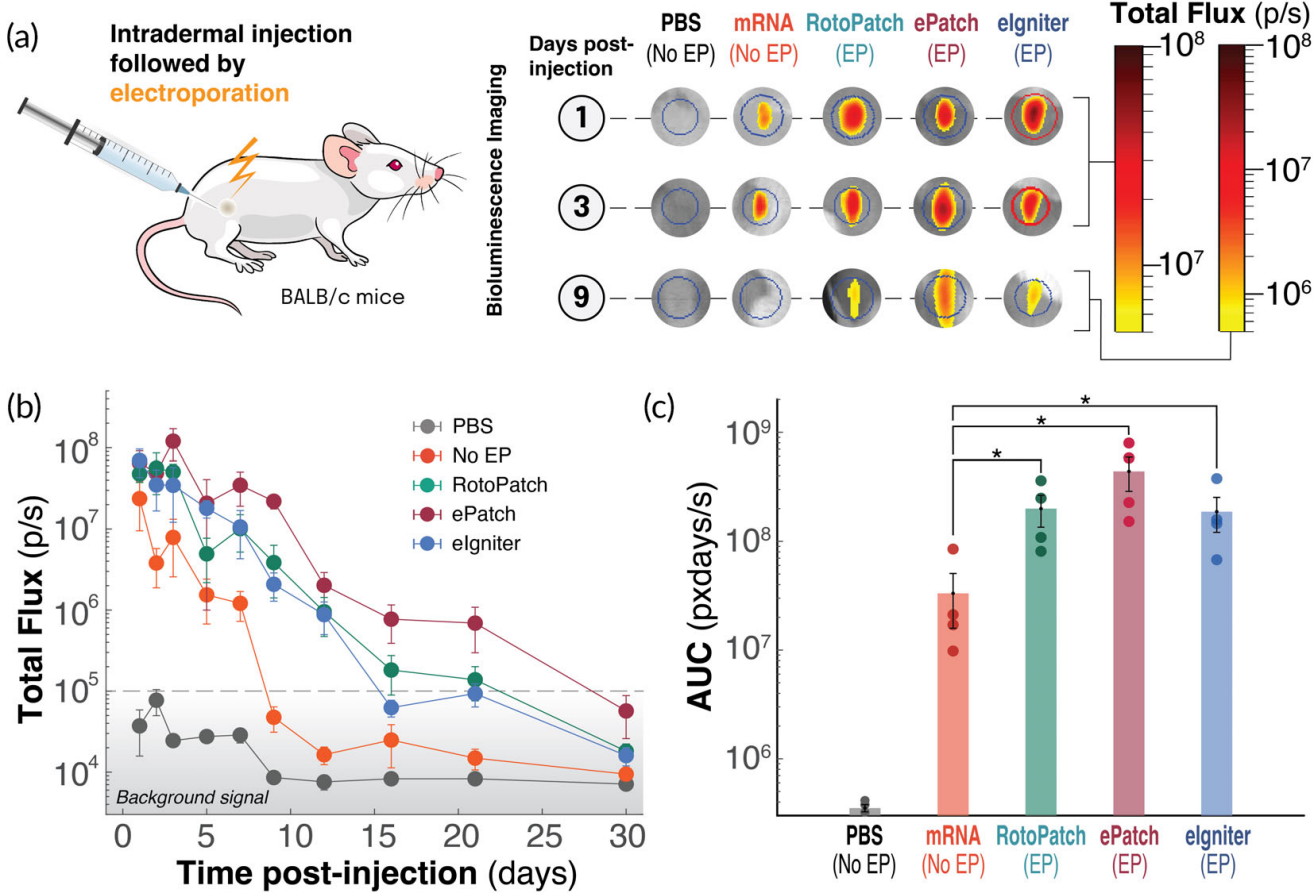
Effect of intradermal electroporation via RotoPatch, ePatch, and eIgniter on in vivo expression of firefly‐luciferase reporter mRNA in mice. (a) Protocol schematic and representative bioluminescence images of mouse skin intradermally injected with 5 μg of naked mRNA (in 20 μL) followed by electroporation (EP) using RotoPatch, ePatch, or eIgniter, as well as control groups without electroporation. The bioluminescence signal was imaged at multiple time points as a measure of in vivo mRNA expression. (b) Kinetics of total bioluminescence flux over 30 days post‐injection of mRNA. (c) Area under the curve (AUC) of the total flux curves in part (b), comparing cumulative in vivo expression of mRNA delivered with or without electroporation. Data are presented as mean ± SEM (*n* = 4); *p <0.05.

We next compared the ability of RotoPatch, ePatch, and eIgniter to enhance delivery and expression of N1‐methylpseudouridine‐modified firefly luciferase mRNA in the skin of mice after intradermal injection of naked mRNA followed by application of 10 electric pulses (Figure 3a). Electroporation by all three devices significantly enhanced reporter protein (i.e., luciferase) expression compared to mRNA injection without electroporation (*p* <0.05, Figure 3b). At 24 h post‐injection, luciferase expression was boosted 2 to 4‐fold across all electroporation groups compared to the non‐electroporated mRNA group. While protein expression in the absence of electroporation rapidly declined, dropping an order of magnitude by day 5 and reaching background levels by day 9, electroporation by any of our three methods sustained significantly higher expression levels for longer durations. Electroporation by eIgniter supported expression for up to 16 days, while RotoPatch extended expression to ~21 days, and ePatch showed the most prolonged effect, with detectable expression lasting for at least 3 weeks, returning to baseline by day 30 (Figure 3b).

To quantify total expression over time, we calculated the area under the curve (AUC) of the luciferase flux profiles. The piezoelectric devices (RotoPatch and ePatch) significantly enhanced cumulative protein expression compared to no electroporation (*p* <0.05, Figure 3c), exhibiting a 6‐fold increase for RotoPatch and a 13‐fold increase for ePatch. Electroporation‐mediated mRNA delivery via eIgniter also resulted in a significant increase in cumulative expression relative to mRNA delivery without electroporation (*p* <0.05, Figure 3c). Among the three electroporation devices, there was no significant difference in their cumulative protein expression over 30 days (*p* >0.05, Figure 3c).

Overall, these findings highlight the potential of our multi‐pulse electroporators for carrier‐free enhancement of mRNA delivery to cells in the skin, enabling sustained protein expression.

### 3.4 RotoPatch enhancement of intradermal plasmid DNA expression

**FIGURE 4:**
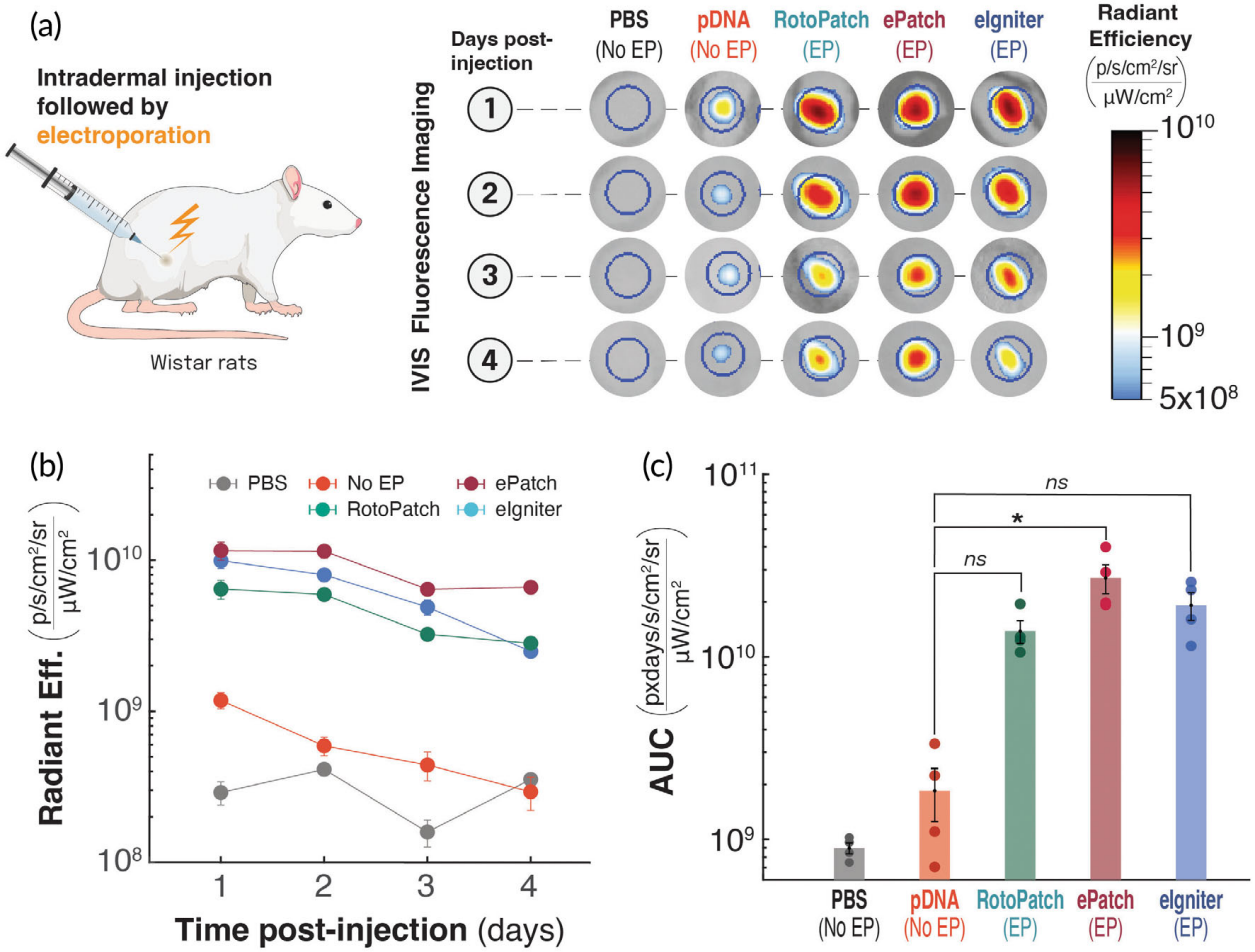
Effect of intradermal electroporation via RotoPatch, ePatch, and eIgniter on in vivo expression of GFP reporter plasmid in rats. (a) Protocol schematic and representative fluorescence images of rat skin injected intradermally with 50 μg of naked plasmid DNA (in 20 μL) followed by electroporation (EP) using RotoPatch, ePatch, or eIgniter, as well as control groups without electroporation. Fluorescence signal was imaged at multiple time points (1–4 days post‐injection) as a measure in vivo DNA expression. (b) Kinetics of radiant frequency over 4 days post‐injection of DNA. (c) Area under the curve (AUC) of the total flux curves in part (b), comparing cumulative in vivo expression of DNA delivered with or without electroporation. Data are presented as mean ± SEM (*n* = 4); *p <0.05, ns = not significant.

Because electroporation is also of interest to delivering DNA into cells, we next tested the transfection capabilities of RotoPatch, ePatch, and eIgniter to deliver a GFP‐encoded plasmid DNA to cells in the skin after giving a series of 10 electric pulses (Figure 4a). Twenty‐four hours after treatment, plasmid DNA alone produced only faint GFP fluorescence, and injection of PBS alone produced background levels of signal (Figure 4b). Electroporation with the RotoPatch, ePatch, or eIgniter boosted reporter protein (i.e., GFP) expression by roughly an order of magnitude compared with the non‐electroporated plasmid site (Figure 4b). Reporter fluorescence from plasmid injection without electroporation fell to baseline within 3 days, whereas electroporated sites retained elevated expression until the end of the experiment on day 4 (Figure 4a). On day 4, the ePatch sustained the highest GFP signal, followed by the RotoPatch and, lastly, the eIgniter (*p* <0.05, Figure 4b). Quantitatively, day‐4 radiance dropped by 61.5% ± 12.3% for the ePatch, 55.9% ± 28.4% for the RotoPatch, and 30.4% ± 20.6% for the eIgniter relative to their respective day‐1 mean radiant efficiency (Figure 4b).

Over the 4‐day period, cumulative GFP expression (AUC) for sites electroporated by ePatch was significantly greater (~13‐fold) than at sites that received plasmid without electroporation (*p* <0.05; Figure 4c). The difference between GFP expression from plasmid delivery with RotoPatch (~6.6‐fold) or eIgniter (~8.5‐fold), and delivery without electroporation was not significant (*p* >0.05). Moreover, there was no statistical difference in GFP expression among the three electroporation devices (*p* >0.05; Figure 4c).

## 4 DISCUSSION

This study introduced a novel approach to electroporation using low‐cost, hand‐held electroporators for simplified nucleic acid delivery in skin that avoids the complications of carrier‐mediated delivery by LNPs, viral vectors, and other approaches. Building off our previous work on ePatch, which provides individual electric pulses after each manual thumb‐click,^30^ the manual and motorized RotoPatch and the eIgniter provide multiple electric pulses after a single manual or motorized rotation or a button‐push, enabling carrier‐free delivery of naked mRNA and plasmid DNA in mice and rats, respectively.

### 4.1 Development of RotoPatch to simplify multi‐pulse electroporation for nucleic‐acid delivery

While ePatch ushered in a new approach to skin electroporation, its translation was limited by the need to apply multiple, manual thumb activations in order to administer multiple electric pulses.^30^, ^44^, ^47^ To address this operational limitation of ePatch, which requires ~40 N of manual normal force per pulse, we developed RotoPatch, a rotary‐actuated pulser requiring only ~5 N of tangential force. Lower force for operation, along with its ability to generate up to nine pulses per rotation from a single piezoelectric crystal, can enhance operator usability and scalability, making it better suited for routine and large‐scale use by healthcare providers requiring minimal training.

We developed three types of RotoPatches—the manual RotoPatch, manual RotoPatch with handle, and motorized RotoPatch. The manual RotoPatch is the simplest, lowest‐cost design that can administer multiple pulses with a single rotational twist of the hand, like opening the lid of a jar. While simple, this method only administered 3 pulses in this study (although future designs may be able to administer more) and required hand motion that is not easily ergonomic.

The improvement on the manual RotoPatch, we added a foldable handle to the rotation mechanism, thereby providing greater leverage when turning the rotary cap, enabling up to 9 pulses per rotation using a more ergonomic hand and arm motion. The addition of the handle increased RotoPatch size (which was minimized during storage by designing the handle to fold up) and cost, but these small negative impacts are likely to be outweighed by the positive impacts of simplified usability.

A final improvement to the RotoPatch was the use of a small, inexpensive motor to rotate the RotoPatch rotor and apply any number of pulses, according to user input. The motor resulted in a small increase in size, and likely added $2 to the cost, indicating that the motorized RotoPatch may be suitable as a fully disposable device in some markets, but may need to be reusable in low‐resource settings, in combination with a single‐use MEA component that contacts the skin.

We also evaluated a battery‐powered pulse generator with no moving parts (eIgniter) as an alternative electroporation device. The eIgniter is a more sophisticated microelectronic device compared to RotoPatch or ePatch but offers the advantage of pushbutton actuation and microelectronic control in a low‐cost format.

Among the various electroporators in this study, all of them significantly enhanced gene and protein expression following intradermal delivery of mRNA and plasmid DNA, suggesting that RotoPatch, ePatch, and eIgniter could all be useful for applications involving nucleic acid delivery. There were, however, some differences between the strength and duration of protein expression following mRNA and DNA delivery by the different electroporation devices, some of which were statistically significant and others of which were not. Additional research is needed to study transfection and expression using these devices to more fully determine not only the statistical significance of possible differences but also to assess the functional differences for specific applications.

The use of widely available piezoelectric and electrical components, such as those found in barbeque lighters, highlights the potential for rapid, global scalability of these electroporation devices for vaccination, particularly in response to future endemic or pandemic outbreak scenarios. Moreover, the prolonged expression achieved with mRNA delivered via piezoelectric electroporation highlights the promise of this technology in gene therapy applications.

### 4.2 Microseconds‐long oscillatory waveforms for electroporation

While exponential‐decay and square wave pulses have traditionally been the standard for electroporation, sinusoidal bipolar oscillatory waveforms have also demonstrated efficacy in achieving reversible electroporation.^27^, ^30^, ^46^ Millisecond‐long pulses are often used for electroporation, but microsecond‐long oscillatory electric pulses have also been effective, attributed to sonication effects and lower membrane polarization.^28^, ^29^, ^30^, ^31^ Both piezoelectric RotoPatch and ePatch, as well as the microelectronic eIgniter, generated high‐voltage, bipolar oscillatory pulses lasting a few microseconds, although the exact waveforms of the piezoelectric pulsers and the microelectronic pulser were different. To enhance transfection efficiency, we reduced the electrode spacing to 650 μm from 900 μm previously used in ePatch,^30^ which resulted in a higher electric field strength in this study. Both piezoelectric electroporators generated peak electric field strengths of ~3–4 kV/cm in the skin while the eIgniter achieved peak electric field strengths up to ~7 kV/cm.

### 4.3 Comparison of electroporators for gene transfection

Conventional commercial electroporators employ advanced electronics to precisely control pulse parameters across microsecond to millisecond durations, which contributes to their high cost and bulky design (Table 1). In contrast, the handheld electroporators developed in this study leverage readily available components, such as piezoelectric crystals, metallic hammers, springs, and 3D‐printed plastic parts, to enable a low‐cost, scalable, and globally accessible electroporation platform.

**TABLE 1:** Comparison of electroporator devices used in gene transfection.

Electroporator	Cost (USD)	Electric field strength (kV/cm)	Power mechanism	Weight (kg)	Waveform type
BTX AgilePulse	$20,000	0.25–5	Electricity	11.3	Square
Cliniporator	$60,000	1.3–10	Electricity	85	Square
Cellectra	Not disclosed	0.13–0.67	Battery	Not disclosed	Square
*e*Patch	$1	1–3	Manual	0.05	Oscillatory
*e*Igniter	$2	7	Battery	0.14	Oscillatory
RotoPatch (Manual and Motorized)	$5	3–4	Manual (and Battery)	0.05–0.4	Oscillatory

RotoPatch, in particular, offers flexibility for diverse clinical settings: the motorized version facilitates ease of use for providers, while the manual version may be better‐suited for resource‐limited environments. Our findings demonstrate that all three electroporator platforms (RotoPatch, ePatch, and eIgniter) effectively enhance cellular uptake of nucleic acids, providing a cost‐effective and efficient alternative for gene delivery. Furthermore, the electric field strength can be adjusted by modifying the electrode spacing within the MEA, allowing customization for applications such as vaccination, gene therapy, drug delivery, and electrochemotherapy. Additionally, we introduced a battery‐powered, low‐cost electroporator (eIgniter) as an even‐simpler‐to‐use tool for nucleic acid delivery.

### 4.4 Limitations

While RotoPatch, ePatch, and eIgniter can provide simple, low‐cost, and portable nucleic acid delivery, several limitations remain. According to their design, the voltage/electric field strength, duration, and waveform of the pulses from a given device are fixed; their modulation requires hardware changes, such as using a different piezoelectric crystal, applying a different strike force with the hammer, or altering microneedle electrode spacing. These constraints limit flexibility in pulse optimization and mean that some changes may not be possible given the limitations of pulser design. Further optimization is needed to optimize pulser performance while maintaining the simple, low‐cost device design.

The in vivo data in this study were limited to a small animal group (*n* = 4), focusing primarily on expression kinetics in mice and rats rather than therapeutic efficacy or immunogenicity. Future studies should evaluate vaccination potential against specific infectious diseases and the therapeutic potential of drugs enabled by electroporation via these devices in larger animal models and eventually in humans. Data were also generated by expert researchers in a laboratory environment. Future studies need to assess usability by clinicians in various healthcare settings.

Although the ePatch has previously demonstrated good tolerability in human subjects, the new device designs, the different waveform of the eIgniter, and higher electric field strengths generated by these devices (due to tighter electrode spacing in the MEA) warrant further assessment of safety and tolerability in human subjects. Future efforts should also aim to integrate drug delivery and electroporation into a single platform for one‐step delivery that avoids conventional intradermal nucleic acid injection followed by application of electric pulses using a separate device.

## 5 CONCLUSIONS

This study presents the development of low‐cost, handheld electroporators capable of administering multiple pulses by a single manual, motorized, or electronic activation for nucleic acid delivery. ePatch was shown in this study and previously to be effective for nucleic acid delivery but required repeated manual activation to produce multiple electric pulses. We developed RotoPatch, a low‐cost, rotary‐actuated piezoelectric electroporator that was able to administer up to 3 pulses during unassisted manual operation, up to 9 pulses during handle‐assisted operation, and effectively unlimited pulses during motorized operation after a single operator actuation. eIgniter also enabled effectively unlimited pulses with no moving parts after a single pushbutton actuation. All pulsers were similarly effective for intradermal delivery and expression of mRNA and plasmid DNA in mice and rats, respectively. RotoPatch and eIgniter enhance transfection efficiency without the cost and complexity of conventional electroporation devices or of carrier‐mediated delivery systems like LNPs and viral vectors. By generating multiple high‐voltage pulses per rotation rather than the individual pulses produced by ePatch, RotoPatch, and eIgniter are designed to reduce user fatigue while still enabling simplified use in clinical environments and resource‐limited settings. These findings highlight the potential of low‐cost, handheld electroporators for scalable, carrier‐free delivery of RNA and DNA.

## AUTHOR CONTRIBUTIONS

*Conceptualization*: PR, EA, SB, and MRP. *Methodology*: PR, EA. *Investigation*: PR, EA, SP, and AL. *Device design and fabrication*: EA, PR, SB, and MRP. *Image data analysis*: PR, SB, and MRP. *Funding acquisition*: PR, SB, and MRP. *Supervision*: SB and MRP. *Writing, original draft*: PR and EA. *Writing, review and editing*: All authors.

## CONFLICT OF INTEREST STATEMENT

MRP is an inventor of patents, consultant to companies, and founder and shareholder of companies that are developing microneedle patch technologies related to those addressed in this study. SB is an inventor of patents and a founder and shareholder of a company that is developing electroporation technologies related to those addressed in this study. These conflicts of interest are managed by Georgia Tech. The other authors have no conflicts of interest to report.

## Supporting information


**Data S1:** Supplementary Data

**Supplementary Video S1:** Demonstration of the motorized RotoPatch in operation.

**Data S2:** Supplementary Information

## Data Availability

All data and materials used in this study are provided in the main manuscript, the supplementary information, and a supplementary file.
