# Supplementary material for: Low‐cost, handheld, multi‐pulse electroporators for simplified nucleic acid delivery in skin
Source: Bioeng Transl Med. 2025 Sep 8;11(1):e70070. doi: 10.1002/btm2.70070 (PMC12821207; doi:10.1002/btm2.70070)
Supplement: Supplementary file 3 — Data S2: Supplementary Information [file BTM2-11-e70070-s001.pdf]

Supplementary Information

Low-cost, handheld, multi-pulse electroporators for simplified nucleic acid delivery in skin

Pankaj Rohilla[1,*], Erkan Azizoglu[1,*], Sion Park[1], Atharva Lele[1], Mark R. Prausnitz[1,✉], Saad Bhamla[1,✉]

[1]School of Chemical & Biomolecular Engineering, Georgia Institute of Technology, Atlanta, GA, USA
[*]Equal contribution.
✉Corresponding author: prausnitz@gatech.edu, saadb@chbe.gatech.edu

ARRIVE Guidelines

The ARRIVE guidelines for our animal studies are as follows:

1. *Study design*: We reported all control and experimental groups in our study.
2. *Sample size*: We have included this information in the paper for different in vivo experiments.
3. *Inclusion and exclusion data*: The inclusion requirement was healthy female BALB/c mice age 7 – 18 weeks or healthy female Wistar rats age 6 – 8 weeks. There were no exclusion requirements.
4. *Randomization*: We used the animals from the cages received from animal vendor (Charles River, Wilmington, MA). The cages were chosen randomly for a specific experimental group and the animals within the cages were also chosen randomly,
5. *Blinding/masking*: There was no blinding or masking in this study.
6. *Outcome measures*: We reported the outcome measures of the study using gene expression for plasmid DNA data and protein expression for mRNA data.
7. *Statistical methods*: All statistical methods used in this study are reported in the "Statistical analysis" subsection of the "Materials and Methods" section.
8. *Experimental animals*: All information of the animals used in the studies is reported, including the species, strain, sex, and age.
9. *Experimental procedures*: All experimental procedures used in our study are reported in the manuscript.
10. *Results*: We presented summary statistics for each experimental group, including their variability as indicated by the standard error of the mean (SEM).

Table S1. List of components used in fabrication of RotoPatch

**Item**	**Specification**
**Motor**	NEMA 17 Stepper HT (LDO Motors)
**Arduino/Driver**	Arduino Uno / DRV 8825
**Stepper Controller**	ZK-SMC02
**3D printed assembly**	Rotary cap, rotor, hammer assembly, stator, MEA

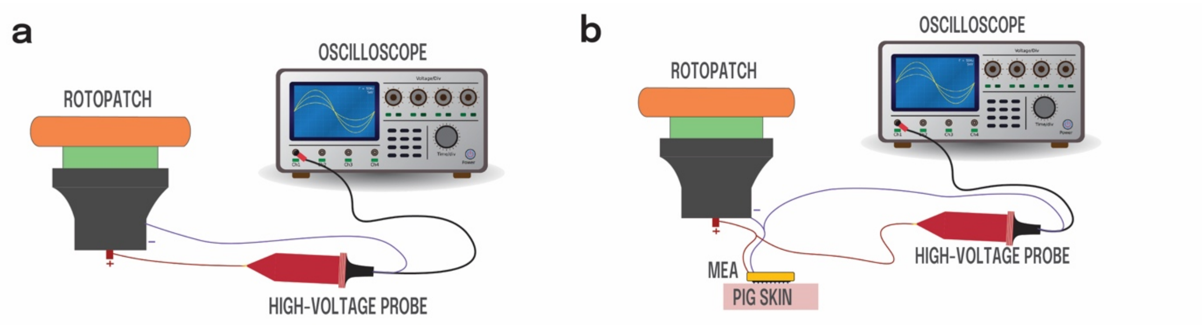

Figure S1. Electrical characterization of RotoPatch. a) Open-circuit setup for measuring voltage profile during RotoPatch actuation. **b)** Closed-circuit setup for measuring voltage profile during electric discharge in ex vivo porcine skin delivered via a RotoPatch.

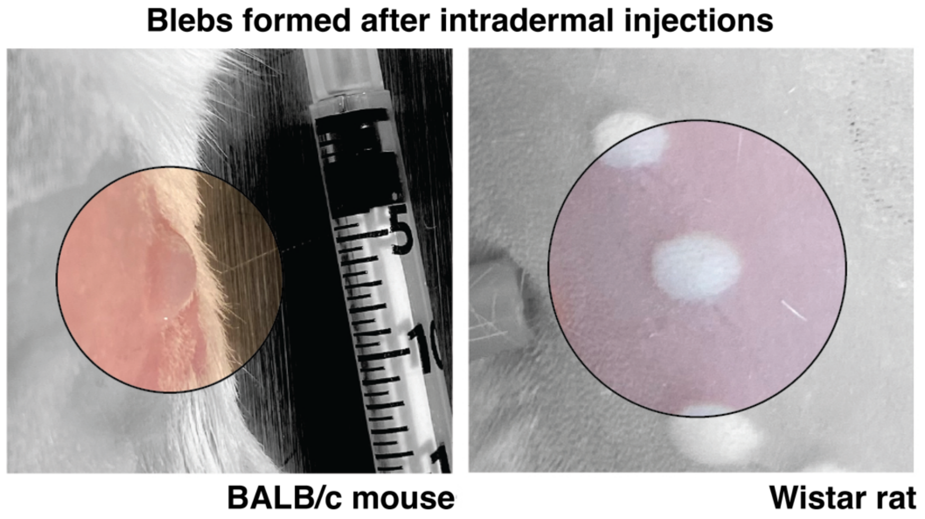

Figure S2. Representative blebs formed following intradermal injections in BALB/c mouse (left) and Wistar rat (right). Circular insets show regions of skin in color where blebs were formed.

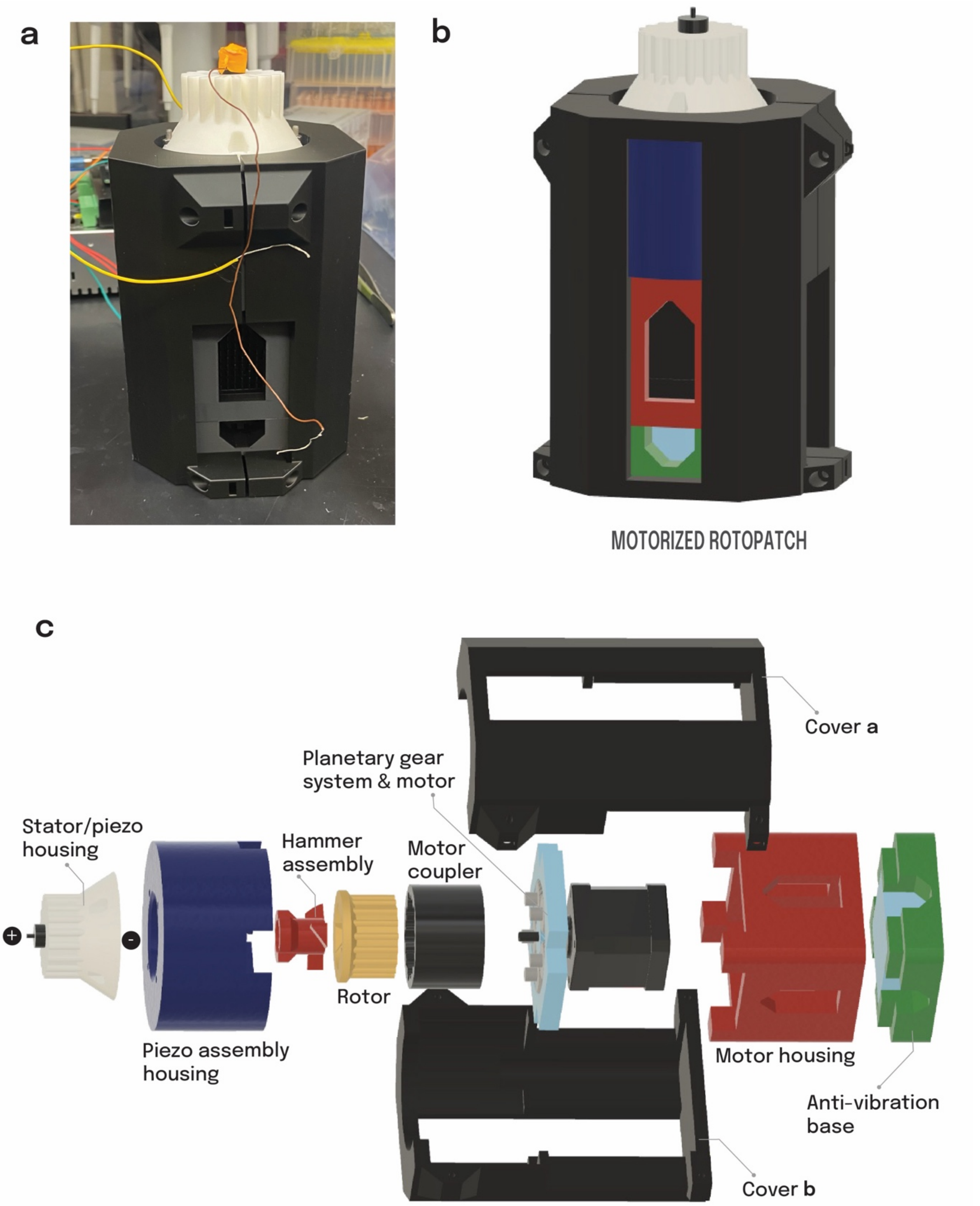

Figure S3. Motorized RotoPatch design used for in vivo studies. a) Fully assembled motorized RotoPatch**. b)** Schematic image of motorized RotoPatch. **c)** Expanded view of the motorized RotoPatch showing different components.

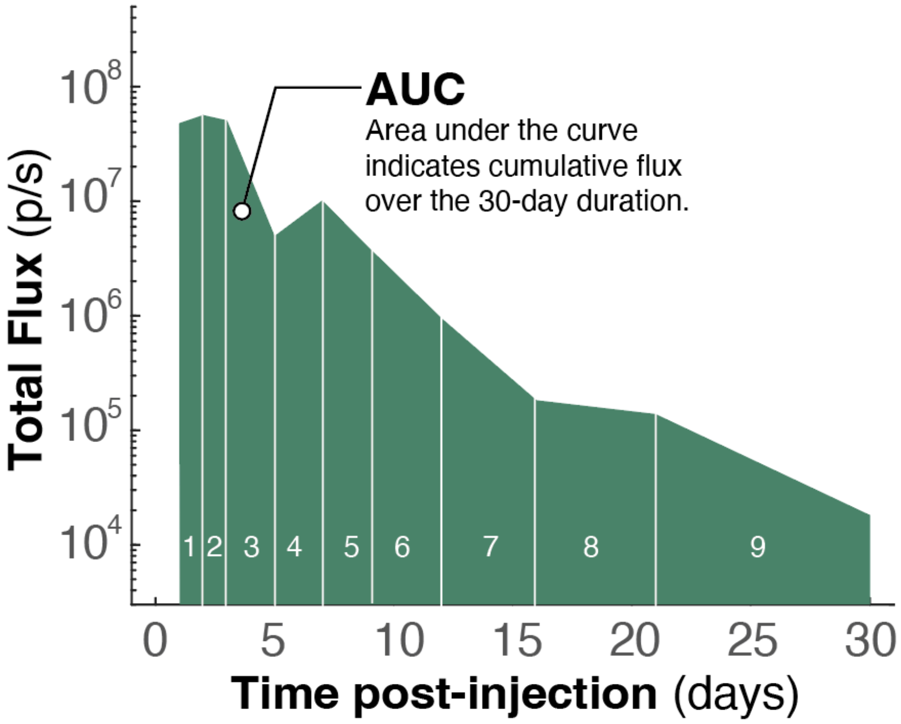

Figure S4. Measurement of the total flux over a 30-day period, quantified as the area under the curve (AUC) of protein expression of fluc-mRNA delivered in BALB/c mice using a RotoPatch.

We used the trapezoidal rule (using the trapz function in MATLAB) to numerically approximate the area under the curve (AUC) by partitioning it into nine trapezoidal segments (Figure S7). Since the data points were unevenly spaced, the trapezoidal rule accounted for variable intervals using the following formula to estimate the area under the curve:

$$\int_a^b f(x)dx \approx \frac{1}{2}\sum_{n=1}^{N}(x_{n+1} - x_n)[f(x_n) + f(x_{n+1})]$$

Where $a = x_1 < x_2 < \ldots x_N < x_{N+1} = b$, and $(x_{n+1} - x_n)$ is the spacing between each consecutive pair of points.

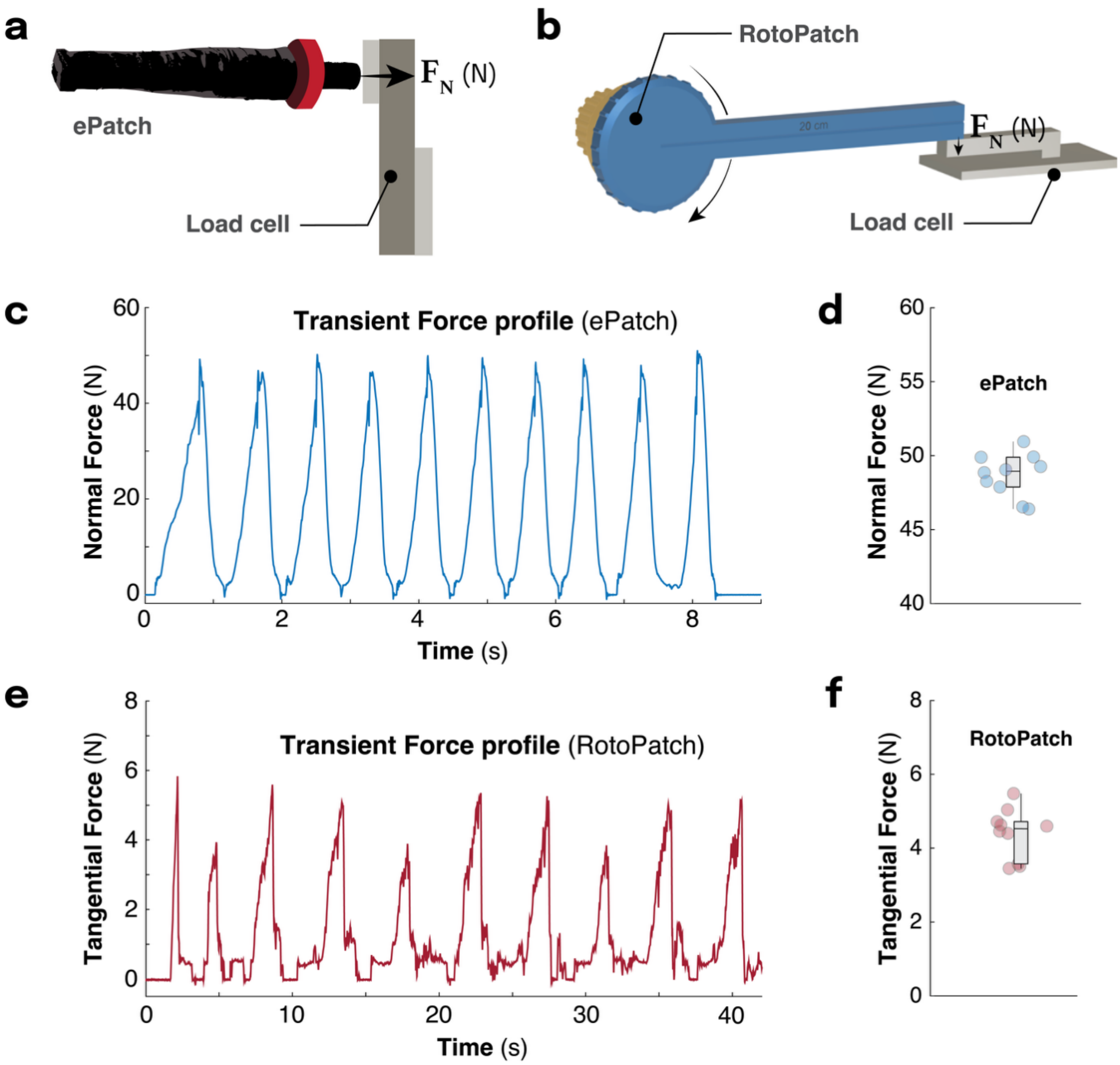

Figure S5. Force characterization of piezoelectric pulsers. a) Schematic of the setup for measuring normal force applied to achieve ePatch actuation. **b)** Schematic of the setup for measuring tangential force applied to achieve RotoPatch actuation. **c)** Transient normal force profile recorded over 10 manual clicks of the ePatch pulser. **d)** Peak normal force values for 10 ePatch actuations for generation of electric pulses (n = 10). **e)** Transient tangential force profile recorded over 10 rotational actuations of the RotoPatch pulser**. f)** Peak tangential force values for generation of pulses during RotoPatch actuation (n = 10).

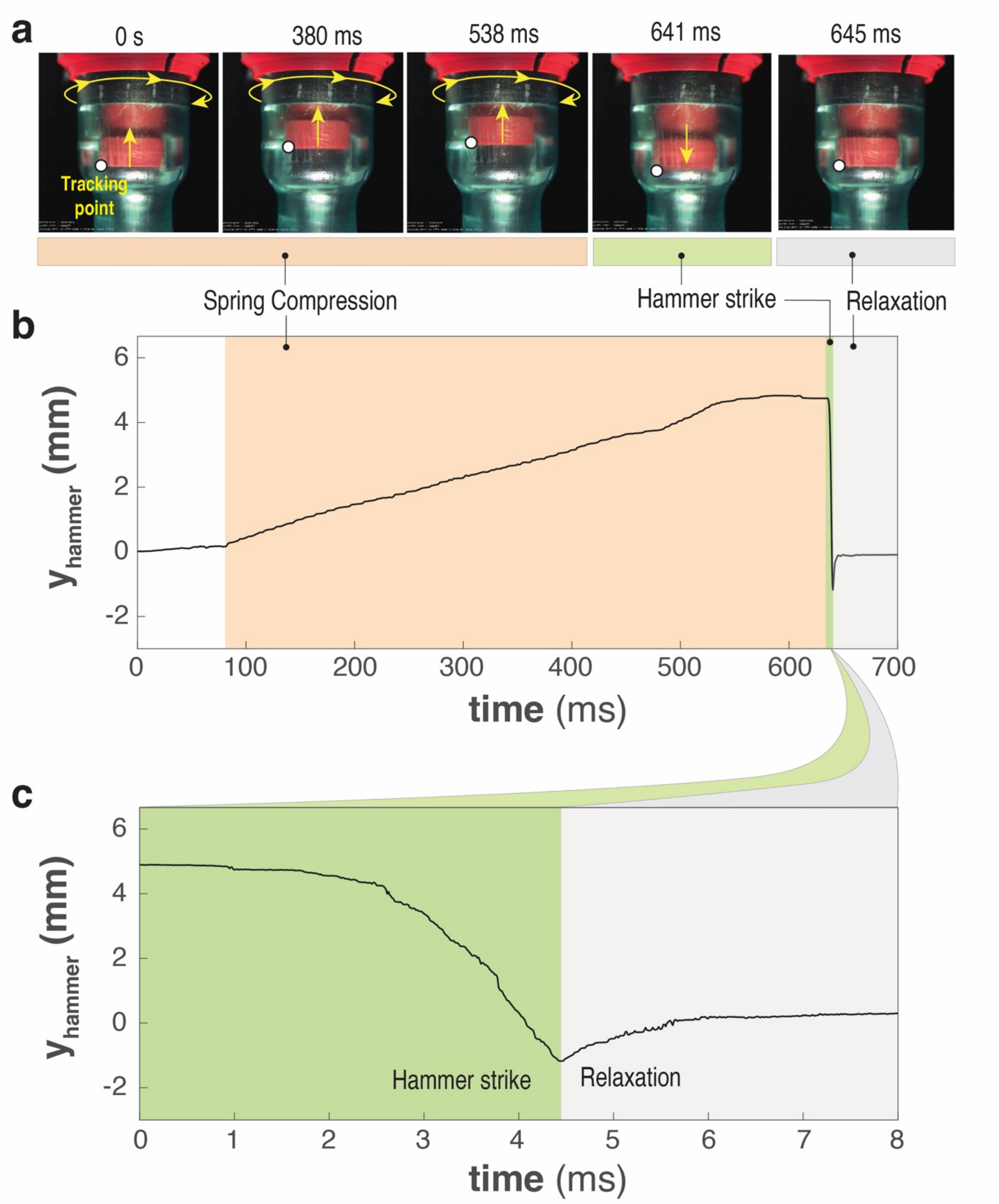

Figure S6. Mechanical behavior of RotoPatch during pulse actuation. a) Snapshots from a high-speed video showing actuation of a single pulse from the RotoPatch. As the planetary gear system drives the rotor, the rotor's interfacing ridges lift the hammer, which in turn compresses the spring (0-76 ms). Immediately after being released from the rotor's ridge, the hammer impulsively moves downward, striking the piezoelectric crystal (76.1 ms), to gene an electric pulse. **b)** Complete trajectory of the hammer during generation of an electric pulse. **c)** Inset from panel b) showing the vertical displacement of the hammer, showing the hammer strike and relaxation events.

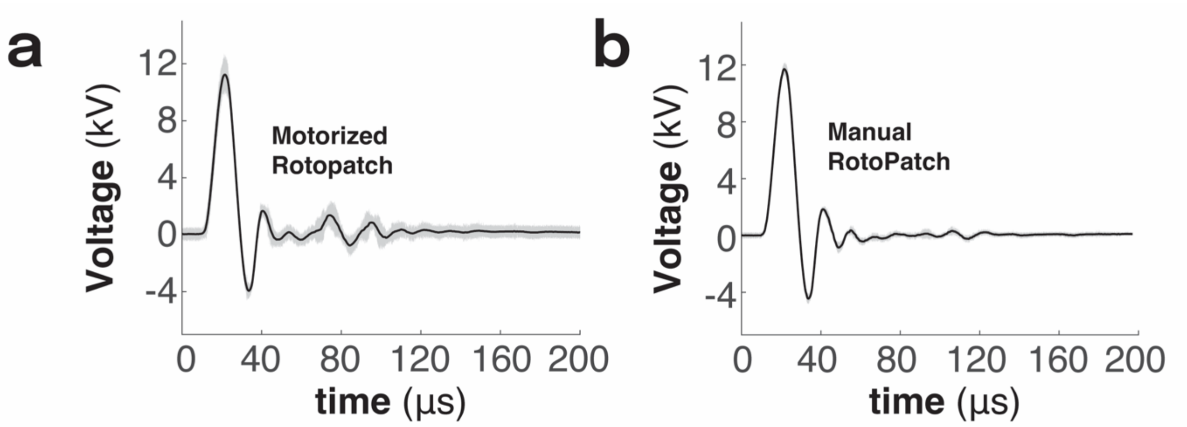

Figure S7. Voltage measurement for motorized and manual RotoPatch shown in Figure 1. a) Open-circuit voltage discharge measurements made using an oscilloscope **a)** for a motorized RotoPatch. and **(b)** for a manual RotoPatch. Shaded error bars represent mean $\pm$ SEM (n = 5).
